# Cross-sectional longitudinal study of the academic half-day format in a hematology-oncology fellowship training program

**DOI:** 10.1186/s12909-015-0418-y

**Published:** 2015-08-25

**Authors:** Ahmed Eid, Peggy Hsieh, Pankil Shah, Robert Wolff

**Affiliations:** 1 Department of General Medical Oncology, Unit 0462, The University of Texas MD Anderson Cancer Center, 1515 Holcombe Blvd., Houston, TX 77030 USA; 2 Department of Internal Medicine, The University of Texas Medical School at Houston, 6431 Fannin, Suite JJL 302, Houston, TX 77030 USA; 3 Department of Infectious Disease & Infection Control Research, The University of Texas MD Anderson Cancer Center, Houston, TX USA; 4 Department of Gastrointestinal Medical Oncology, UT MD Anderson Cancer Center, Houston, TX USA

## Abstract

**Background:**

Few published studies have evaluated the effectiveness of changing the traditional curriculum of several hourly educational sessions per week to an academic half-day (AHD) educational format. This study describes our experience with implementing an AHD format in the Hematology-Oncology Fellowship Program at The University of Texas MD Anderson Cancer Center and evaluates the perceptions that learners had for this format.

**Methods:**

Using a mixed quantitative and qualitative approach, we evaluated our AHD program using four yearly fellows’ surveys to assess the Hematology-Oncology Fellows’ perceptions of the effectiveness of the AHD format. We analyzed the fellows’ perceptions using descriptive statistics and thematic analysis of the qualitative data collected from the surveys. We used a quality improvement approach by implementing and testing changes to the AHD over 4 years on the basis of the data collected from the yearly fellows’ surveys. We also collected third-year fellows’ Oncology In Training Exam (ITE) scores from 2008 to 2014.

**Results:**

We found that the fellows perceived the AHD format favorably; fellows agreed that they had more motivation to attend AHD, more concentration during the sessions, more effective weekly work organization, and increased knowledge retention. We established the reliability of our survey tool (Cronbach’s alpha = 0.83) as well as content and construct validity. We saw an increasing trend in ITE scores since the AHD was implemented.

**Conclusions:**

Our results contribute to further understanding the effect of the AHD format on trainees. Using a continuous evaluation and an educational quality improvement strategy, we found that the AHD curriculum was associated with a rising trend in learners’ exam scores and increased learner satisfaction.

**Electronic supplementary material:**

The online version of this article (doi:10.1186/s12909-015-0418-y) contains supplementary material, which is available to authorized users.

## Background

To our knowledge, no studies have examined the effectiveness of the academic half-day (AHD) format in internal medicine subspecialty fellowship programs. To the best of our knowledge and according to recent publications [1], no available validated survey exists to evaluate trainees’ perceptions about their AHD experience. To address this gap in the literature, we present our experience and evaluate the evolution of the AHD format at The University of Texas MD Anderson Cancer Center Hematology-Oncology Fellowship Program and the validation of our survey tool.

The Accreditation Council for Graduate Medical Education mandates that internal medicine graduate medical programs must schedule regular didactic sessions that include lectures and other media that communicate the core principles of internal medicine [2]. Traditionally, many programs have fulfilled this requirement with noon conferences or other 1-h sessions several days a week. Evidence about whether these didactic lectures in internal medicine residencies lead to long-term knowledge retention varies [3–5], and some studies suggest that resident performance is not necessarily enhanced by this educational structure [6]. Currently, some training programs have altered the format and combined multiple sessions per week into one AHD session per week. This shift away from the traditional model could enhance knowledge acquisition and influence clinical performance, according to previous research on the AHD format [7].

However, few residency programs have provided descriptions of their AHD-format curricula [7–13] or evidence of effectiveness [1, 14, 15]. Publications about the implications of the AHD format and the opinions of the trainees in these programs as well as validated methods to measure the effectiveness and usefulness of the AHD format are lacking. Such measurement is critical for program evaluation, improvement, and reliable comparison among the approaches employed by various institutions or by the same institution over time.

This study describes the implementation and evolution of our AHD program, the perceptions of the fellows as the program evolved, and the improvement strategies developed on the basis of fellows’ evaluations and feedback.

## Methods

### AHD description

**Fig. 1:**
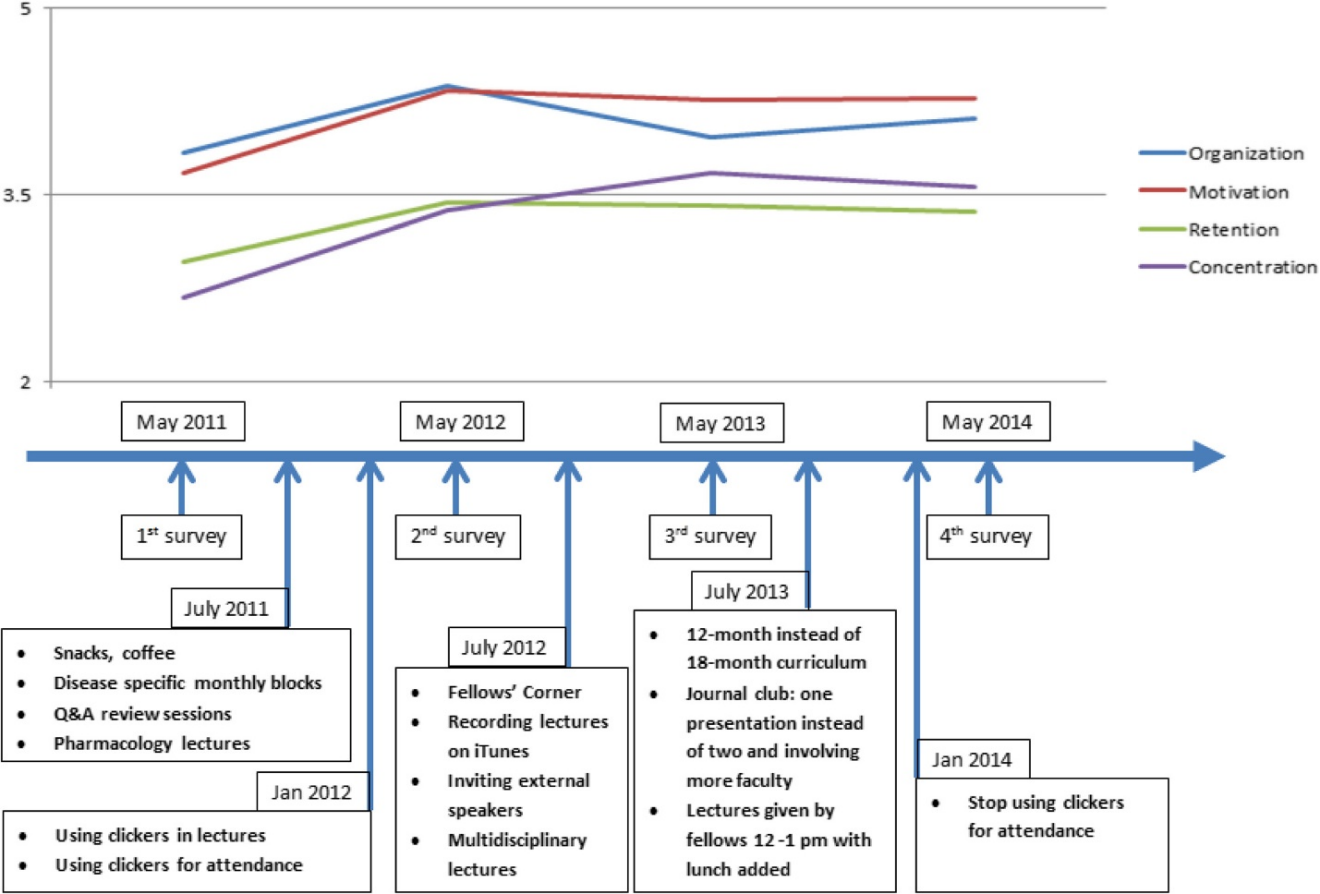
The Perception of the Fellows of the Academic Half-Day Format Over Four Years and of the Implemented Changes

In July 2009, the Hematology-Oncology Fellowship Program changed the daily noon conference format to a weekly AHD format. The new format consists of a single half-day (3–4 h) each week. This half-day session is held every Tuesday morning after a Grand Rounds seminar and consists of a group meeting with the Grand Rounds seminar’s chief lecturer (Fellows’ Round-Table) or a session presented by a fellow (Fellows’ Corner), followed by two lectures given by attending physicians. In July 2013, the sessions presented by fellows were moved to noon, with an additional hour during some weeks. Our program communicated with faculty in various departments to ensure that the fellows would be excused from their clinical and research responsibilities to attend the AHD sessions. On the basis of feedback received via the yearly survey of the fellows, we provided additional services and implemented changes in the AHD. These improvements are listed in Fig. 1.

### Procedures

In May of each academic year from 2011 through 2014, fellows enrolled in the program were asked to complete a paper-based survey about their perception of the AHD format. For the first year of the study (2011), in addition to the currently enrolled fellows, the fellows who graduated in 2010 were also asked to complete a similar survey anonymously. Thus, a total of seven classes participated in our surveys: the graduating class of 2010 through the class of 2016. The fellows who graduated in 2010, 2011, and 2016 only filled out one survey. The graduating classes of 2012, 2013, 2014, and 2015 participated by filling out the survey more than once.

The surveys consisted of two sections (Additional file 1: Appendix 1). The first section of the survey covered the fellows’ perceptions of their ability to concentrate during the AHD sessions, their motivation to attend the AHD sessions, their retention of information presented during the AHD sessions, and their ability to organize their work schedules around the AHD sessions. The perception survey was a 5-point Likert-like scale asking fellows to rate their perceptions from favorable (anchored at 5) to unfavorable (anchored at 0). In addition to these original four categories, starting in 2013, we included four new items: distraction, usefulness, influence, and refresh (Additional file 1: Appendix 2). The second section of the survey for all four years requested input on the services added to enhance the AHD experience as well as suggestions for improvement.

We collected third-year fellows’ oncology In Training Exam (ITE) scores from 2008 to 2014 to explore the effect of the AHD on the fellows’ cognitive knowledge acquisition since the program was implemented. The oncology ITE is a validated, standardized examination aimed at assessing oncology fellows’ medical knowledge and identifying areas of deficiencies that require further learning intervention.

### Statistical analysis

We conducted all analyses using Statistical Package for the Social Sciences software, version 21. We ran descriptive analysis to describe the sample distribution over the 4 survey years. We calculated means and standard deviations of the core perception survey items. We carried out multivariate analysis of variance (MANOVA) to examine group differences over the 4 years for four perception items that measured motivation, concentration, organization, and retention. The independent variable was the survey year, and we treated the four perception items as dependent variables.

The University of Texas at MD Anderson Cancer Center Institutional Review Board approved the study (Protocol DR11-0150). Because information was collected anonymously and because participants could not be linked to the data, the study was approved as exempt research, and written consent was not required. All participants read and acknowledged a consent statement explaining the goals and methods of the study and how their privacy and confidentiality would be protected.

## Results

Cronbach’s alpha was carried out to test reliability (r = 0.83). The response rates ranged from 72 to 86 % for the 4 years. The distribution by class of graduation and survey year is presented in Table 1.

**Table 1:** Hematology-Oncology Fellows who participated in the surveys (2011–2014)

Survey year	Survey Participants (total enrolled)	Participation rate	Graduation year (cohort)	Survey Participants (total enrolled)
2011	38 (53)	71.7 %^a^	2010	6 (14)
			2011	14 (16)
			2012	8 (11)
			2013	10 (12)
2012	32 (37)	84.5 %	2012	10 (11)
			2013	11 (12)
			2014	11 (14)
2013	34 (41)	82.9 %	unspecified	5
			2013	8 (12)
			2014	9 (14)
			2015	12 (15)
2014	36 (42)	85.7 %	unspecified	5
			2014	8 (13)
			2015	11 (15)
			2016	12 (14)

^a^ The participation rate for 2011 survey excluding the already graduated fellows was 82 %

We saw an increased acceptance of the AHD format over the 4 years in the areas of organization, motivation, retention, and concentration. The overall mean was 3.70 on the 5-point Likert scale; on average, fellows rated their ability to organize their work schedules around the AHD sessions at 4.05, their motivation to attend the AHD sessions at 4.13, their retention of information presented during the AHD sessions at 3.29, and their ability to concentrate during the AHD sessions at 3.31. Overall, the fellows found the AHD program to be above average. To examine whether the four areas (organization, motivation, retention, and concentration) differed by year, we conducted MANOVA. Significant differences were found among the four years for the dependent variables (Wilks’ λ = .75; *F* (12,352) = 3.42; *P* < 0.001). Analyses of variance (ANOVA) on the dependent variables were conducted as a follow-up test to the MANOVA. Using the Bonferroni method, each ANOVA for each dependent variable was tested. The ANOVA on the four areas are as follows: organization, *F* (3, 136) = 2.55, *P* = 0.06; motivation, *F* (3, 136) = 4.96, *P* < 0.01; retention, *F* (3, 136) = 2.69, *P* < 0.05; and concentration, *F* (3, 136) = 10.06, *P* < 0.001. Post hoc analyses to the univariate ANOVA indicated the greatest difference between the 2011 and 2014 means for the four areas. Results are displayed in Table 2.

**Table 2:** Hematology-Oncology Fellows’ perception of the academic half-day format by survey year (2011–2014)

Perception variable	Year	Mean (SD)	Significance
Organization	2011	3.84 (.95)	*P* = 0.06
	2012	4.38 (0.71)	
	2013	3.97 (0.80)	
	2014	4.11 (.85)	
Motivation	2011	3.68 (1.01)	*P* < 0.01
	2012	4.34 (0.65)	
	2013	4.26 (0.86)	
	2014	4.28 (.74)	
Retention	2011	2.97 (0.99)	*P* < 0.05
	2012	3.44 (0.76)	
	2013	3.41 (0.74)	
	2014	3.36 (.64)	
Concentration	2011	2.68 (0.96)	*P* < 0.001
	2012	3.38 (0.75)	
	2013	3.68 (0.81)	
	2014	3.56 (.84)	

Of the improvements, the organization of the lecture topics in blocks, the inclusion of question and answer sessions, and the provision of snacks were the top three items that helped the fellows receive the most benefit from the AHD sessions. In the 2013 survey, the mean ratings of these three items were 4.42, 4.42, and 4.39, respectively. The means and standard deviations for all items are presented in Table 3. The most common barriers to receiving the maximum benefit from the AHD sessions were calls regarding patient care, research demands, and faculty pressure to fulfill service obligations.

**Table 3:** Means (standard deviation) of fellows’ perception of improvements added to the academic half-day format based on 2013 survey results

Service/incentive	Mean (SD)
Snacks	4.39 (0.62)
Clickers by lecture	3.94 (0.93)
Clickers for attendance	3.33 (1.08)
Arranging lectures by themes	4.42 (0.56)
Q/A sessions	4.42 (0.66)
Pharmacology	4.03 (0.85)
Fellows’ Corner	3.39 (1.03)
Fellows’ Round-Table	3.03 (1.21)
Podcast	3.39 (1.32)

*Abbreviations*: SD standard deviation, *Q/A* question and answer

The qualitative data showed a similar increased acceptance of the AHD format and the implemented improvements among the fellows over the 4 years. This was seen in the following fellows’ comments on the surveys:

*“This academic year is much better than last year. I really enjoy the protected time. If these were lunch-hour lectures, you would have inevitably been late or missed lectures due to patient care. Lectures are now clinically relevant and geared toward the boards!”* [2012 survey]

*“Some lectures can be more board-oriented. Pharmacology lectures are an excellent addition! [This program is a] great way to learn oncology, and it has improved significantly since last year!”* [2012 survey]

*“Sometimes time is not managed well, and gaps are present between lectures. However, there has been a substantial positive improvement since last year.”* [2012 survey]

*“[The AHD] has gotten better over the years.”* [2013 survey]

*“It is so much better compared to last year.”* [2014 survey]

“*I think the 12-month curriculum was a great change. Continue*.” [2014 survey]

Fellows’ comments reflected barriers and opportunities for further improvement:

*“Certain services and rotations make it difficult to learn and benefit from academic Tuesday.”* [2011 survey]

*“I feel that some attendings were upset that we were missing so much clinic time that Tuesday morning.”* [2011 survey]

*“Nurses call all the time, and I cannot sit through an entire lecture without being interrupted.”* [2013 survey]

Cognitive overload was mentioned as one of the main barriers either owing to the length of the didactic session or the content:

*“Most of lectures are didactic and not interactive. It was very difficult to concentrate because of the didactic format.”* [2011 survey]

*“We have to stay focused for a couple of hours, which is also challenging since completion of medical school.”* [2013 survey]

*“Please provide caffeinated soda at lunch. It would make focusing on the noon content easier.”* [2014 survey]

The qualitative results of the fellows’ suggestions for improving the AHD are thematically analyzed and presented in Table 4. The qualitative data from all surveys are listed in Additional file 1: Appendix 3. The survey generation and validation data are listed in Additional file 1: Appendix 4.

**Table 4:** Thematic summary of Hematology-Oncology Fellows’ suggestions for improving the academic half-day format (2011–2014)

	2011	2012	2013	2014
Structure	• Curriculum for sessions	• Curriculum for sessions	• Decrease length of curriculum (18 months to ≤ 12 months)^a^	• Decrease duration from 5 to 4 h
	• Online repository for the lectures	• Website where lectures are held^a^		• More breaks
	• Schedule lectures in blocks (GI, GU, breast, etc.)^a^	• Record Lectures^a^		• Easier access for lectures at home
	• Pre and posttests each month	• References for articles		• Schedule main blocks earlier in year
				• Sign in sheets instead of clickers^a^
Content/Format	• More board review sessions^a^	• Continue board review and pharmacology sessions^a^	• More case-based teaching, perhaps a morning report in residencies to help fellows think more critically	• More clinical lectures
	• Make lectures more interactive	• Make lectures more interactive	• More clinical based talks and board review series	• More interactive sessions
	• More case-based format^a^	• Add different formats: small group, problem-based learning^a^	• More interactive sessions, more varied types of sessions (not just PowerPoint)	• More review questions, more group exercises
	• More pharmacology sessions^a^	• More benign hematology lectures^a^	• More Q&A sessions	• More case-based and problem-based sessions
				• More pharmacology talks
Faculty	• More faculty involvement^a^	• More involvement^a^	• Ensure that best faculty are involved	• Academic leaders participate in lectures
				• Statistics faculty at journal clubs^a^
Learning Environment	• Snacks, coffee breaks^a^	• Consider a room with table set up	• No disturbance from calls and pagers from service	• Reduce the calls from chemo suites during that period
			• More interactive style of learning	

*Abbreviations*: *GI* gastrointestinal, *GU* genitourinary, *Q&A* question and answer

^a^Implemented

**Fig. 2:**
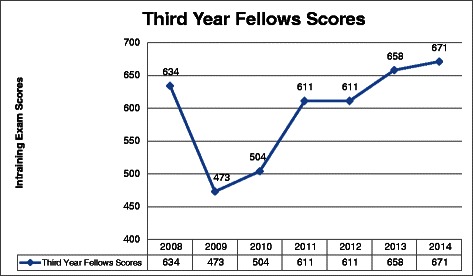
Third-Year Fellows ASCO In training Exam Scores (2008–2014)

Only third year fellows’ oncology ITE scores were complete between 2008 and 2014. We saw an increasing trend in the fellows’ ITE scores since the AHD was implemented (Fig. 2). We were not able to test if this positive trend in ITE scores was statistically significant since we only had access to average collective fellows’ ITE scores and no access to individual scores.

## Discussion

To our knowledge, this is the first study to examine the effectiveness of the AHD format in a fellowship program using an internally validated survey and a quality improvement approach. Although some descriptive articles have been published [7], our study focuses on measuring the fellows’ perceptions of their learning experience. We also showed that the AHD format implemented in our program is well received by the fellows and offers them the opportunity to make the most of this learning experience. The conclusions presented here are based on a 4-year experience with our AHD format and thus offer a more reliable evaluation of the feasibility of this format.

One of the strengths of our study is that the fellows benefited from the improvements that they suggested on our surveys and thus benefited from the study. Our continuous evaluation and improvement approach supports the call for using quality improvement methods in medical education [16].

Another strength of the survey is that we did not restrict it to questions related to how satisfied the fellows were with the AHD format; we also included the effects of the AHD format on secondary and tertiary levels, as defined in the Kirkpatrick model [17]. The survey included questions about organization, motivation, and concentration during the sessions that addressed the first level of the Kirkpatrick model (learner satisfaction). The survey also included questions about how the AHD format helped to refresh fellows’ knowledge and affected their retention of knowledge; these items correlate with the second level of the Kirkpatrick model (learner knowledge). The increasing trend in fellows’ ITE scores since the AHD was implemented (Fig. 2) confirms the fellows’ perception about the improvement in knowledge acquisition after the AHD was implemented. Finally, the survey included questions about how the AHD format influenced decision making in the clinic, which correlates with the third level of the Kirkpatrick model (transfer of knowledge). We hope that future studies will evaluate these questions not only by the fellows’ perceptions but also by other objective and feasible matrices. Our current study serves as a baseline for future research.

In a recently published description of the AHD format in three internal medicine residency programs, the percentage of residents answering “yes” to the Accreditation Council for Graduate Medical Education annual survey question, “Are the core conference series educationally valuable?” was higher in programs with the AHD format than in those with the noon lecture format [7]. Our study’s results support these findings by showing that the AHD format provides an educationally valuable experience for trainees in graduate medical education.

One study that compared knowledge retention differences in the traditional noon or 1-h conference format and the AHD format suggested that a “dispersed delivery” method or the traditional noon conference format was more beneficial for long-term knowledge retention [18]. Another study showed that family practice residents attending didactic lectures in a block conference format did not show improvement in their knowledge over the long-term [15]. However, other studies have found that the AHD format possibly helped improve In-Training Exam scores and had a positive effect on the overall intellectual climate within a residency program [7]. Though we cannot claim causality, ITE scores have improved since the implementation of the AHD, suggesting that the new learning program may have had a positive effect on learning outcomes. The lack of access to individual fellows’ ITE scores prevented us from testing if this trend was statistically significant. Finding a statistically significant difference is also unlikely due to the small sample size of the fellows taking the exam each year.

Therefore, it seems important to continue investigating the AHD format in graduate medical education particularly because our study shows that the fellows perceive this format favorably. Hopefully, further studies will illuminate the effect of the AHD format on knowledge retention and clinical performance.

Our study had several limitations, some of which were inherent to the cross-sectional survey study design. Ensuring that the fellows evaluated the program honestly while in the program could be an inherent conflict of interest for the study. On the one hand, the fellows are the beneficiaries of the teaching program, and their evaluation of the program is thus the most direct measurement of the usefulness and efficacy of the program. On the other hand, they are a vulnerable population, and our institutional review board has strict guidelines to protect the fellows’ interest. To ensure we accounted for both these factors, we decided to conduct this study in a de-identified and anonymous manner.

Another limitation to our study is the lack of information on the fellows’ baseline characteristics and fund of knowledge assessment such as ITE scores during their residency training or at the beginning of the fellowship. This information is important to make sure the increased trend in fellows' ITE scores over the years of the AHD implementation is related to the AHD effect and not due to other variables. Unfortunately this information is not available since our program does not collect residency ITE scores and does not offer the ITE exam to the fellows during the first year of their fellowship training. Although a variation in the fellows’ baseline characteristics is possible, it is less likely that this difference will be significant enough to account for the rise in ITE scores since our program’s selection criteria for new fellows has not changed and the vast majority of our fellows are selected based on their excellent academic track records.

Furthermore, participation was voluntary, and no record was kept of who returned the survey. No identifying questions were asked. Thus, the fellows felt more assured about giving their honest opinion. However, we could not link the data to any other outside variable or even to responses from the previous year(s). This posed the most serious limitation to our study, as we could not corroborate our findings with any data collected outside of the survey. However, we believe that the results we obtained were meaningful and reliable because of the trend among fellows toward more favorable perceptions of the ADH format as we implemented more of their suggestions each year.

It would be worthwhile to evaluate the effect of this new teaching format through more objective outcomes, such as improvement in clinical decision making, and/or improvements in patient care outcomes. However, designing such a study would entail accounting for all the confounding factors related to feasibility and the privacy of the fellows and their patients. Taking that into account, we thought that a voluntary and anonymous estimation of the fellows’ perception was the most direct, unbiased, and feasible method to evaluate the AHD format. Our study adds to the literature on the AHD format, which currently lacks information about the AHD format’s effect on the trainees, training programs, and institutions. We internally validated the core items of the satisfaction survey. Future external validation of the survey in other training programs who have adopted the AHD format is required.

## Conclusions

Using a mixed quantitative and qualitative approach, we used four yearly surveys to assess the Hematology-Oncology Fellows’ perceptions of the effectiveness of the AHD format. We found that fellows perceived the AHD format favorably; fellows agreed that they had more motivation to attend AHD, more concentration during the sessions, more effective weekly work organization, and more knowledge retention. We established the reliability of the survey tool as well as content and construct validity. This study has contributed to understanding the effects of the AHD format on trainees. Using a continuous evaluation and an educational quality improvement strategy, we found that the fellows’ perceptions of the AHD format improved. Though we cannot claim causality, ITE scores have improved since the implementation of the AHD in our program, suggesting that the new learning program may have had a positive effect on learning outcomes. It is important to continue investigating the effect of the AHD format on knowledge retention, concentration, and clinical performance to ensure that this format is beneficial to the learners.

## Additional file

Additional file 1: **Appendices 1 to 4.** (DOCX 305 kb)
