# Supplementary material for: Cross-sectional longitudinal study of the academic half-day format in a hematology-oncology fellowship training program
Source: BMC Med Educ. 2015 Aug 25;15:139. doi: 10.1186/s12909-015-0418-y (PMC4549017; doi:10.1186/s12909-015-0418-y)

**Additional file 1**

**Appendix 1:** 2013 Survey

| Institution |
| --- |
| UT MD Anderson Cancer Center |
| Department/Program |
| Hematology-Oncology Fellowship Program |
| Year of graduation from the program: |
| O 2013 O 2014 O 2015 |
| Anticipated Career Path: |
| O Clinician master/education O Clinician Investigator  O Physician Scientist O Other(specify): |

**Instruction:**

The purpose of this survey is to get your opinion about the Academic Half-Day learning format. The Academic Half-Day consists of a grand round followed by the fellows’ corner and two lectures every Tuesday morning.

**Please circle the response that best describes how you feel about the following items:**

**Core Survey Items**

1) The **organization** of your weekly schedule given the schedule for the Academic Half- Day is …

a) Difficult

b) Slightly difficult c) Moderate

d) Easy

e) Very easy

2) Overall, your **motivation** to attend the Academic Half-Day has been …

a) Very low

b) Below average c) Average

d) Above average

e) Very high

3) Overall, your **ability to retain** the educational material delivered during the Academic

Half-Day has been …

a) Very low

b) Below average c) Average

d) Above average

e) Very high

| 4) Overall, your **ability to concentrate** during the entire time of the Academic Half-Day has been ...  a) Very low  b) Below average c) Average  d) Above average  e) Very high |
| --- |
| 5) D**istractions caused** by your other responsibilities/duties during the course of the  Academic Half-Day has been …  a) Very low  b) Below average c) Average  d) Above average e) Very high |
| 6) How **useful** are the contents presented at the Academic Half-Day to you?  a) Not at all useful  b) Not quite useful  c) Somewhat useful d) Very useful  e) Extremely Useful |
| 7) How often, in your opinion, do the materials taught in the Academic Half-Day,  **influence** your clinical decision making?  a) Very rarely b) Rarely  c) Occasionally d) Frequently  e) Very Frequently |
| 8) How much does the content taught in the Academic Half-Day, **add to/refresh** your knowledge?  a) Not at all  b) Very little c) Somewhat d) Quite a bit  e) To a great extent |

**Qualitative Survey Items**

1) From your experience, what are some barriers for getting the most out of the Academic

Half-day (check all that applies to you):

a) Calls and pages from service

b) Research demands (e.g Lab duties, following cell lines/research animals, meetings, etc …)

c) Calls from patients

d) Calls from chemo suites

e) Faculty pressure to fulfill service obligations (clinic or inpatient wards)

f) There are no barriers g) Other (specify):

2) In your opinion, what are the **Advantages** of the Academic Half-Day?

3) In your opinion, what are the **Disadvantages** of the Academic Half-Day?

4) What are your **Suggestions** for improving the Academic Half-Day?

**The following helped me to get the most out of the Academic Half-day:**

Strongly Disagree Disagree Neither Agree Strongly Agree

Snacks, coffee 🔾 🔾 🔾 🔾 🔾

Using clickers in lectures 🔾 🔾 🔾 🔾 🔾

Using clickers for attendance 🔾 🔾 🔾 🔾 🔾

Disease Specific monthly blocks (Breast, GI,…) 🔾 🔾 🔾 🔾 🔾

Q&A review sessions 🔾 🔾 🔾 🔾 🔾

Pharmacology Lectures 🔾 🔾 🔾 🔾 🔾

Fellows’ Corner 🔾 🔾 🔾 🔾 🔾

Roundtable with Grand Round speakers 🔾 🔾 🔾 🔾 🔾

Recording Lectures on iTunes U 🔾 🔾 🔾 🔾 🔾

**Cognitive Debriefing**

| 1. How easy was it to complete the questions?    1. Difficult    2. Slightly difficult    3. Moderate    4. Fairly easy    5. Very easy |
| --- |
| 1. How comfortable were you in answering the questions?    1. Very uncomfortable    2. Somewhat uncomfortable    3. Neutral    4. Comfortable    5. Very comfortable |
| 1. Rate the completeness of the survey in evaluating all aspects of the Academic Half-Day?    1. Extremely lacking    2. Somewhat lacking    3. Neutral    4. Somewhat comprehensive    5. Very comprehensive |
| 1. How difficult or easy was it to comprehend the questions?    1. Very difficult    2. Slightly difficult    3. Moderate    4. Fairly easy    5. Very easy |

**Appendix 2:** Perception Survey Questions Codes

| **Questions** | **Variable Name**  (as referred to in the manuscript) |
| --- | --- |
| 1. The **organization** of your weekly schedule given the schedule for the academic sessions is … | **organization** |
| 1. Your **motivation** to attend the academic sessions is … | **motivation** |
| 1. Your **ability to retain** the educational material delivered during the academic session is … | **retention** |
| 1. Your **ability to concentrate** during the entire time of the academic session is ... | **concentration** |
| 1. Your level of **distraction** by your other responsibilities/duties during the course of the academic session is … | **distraction** |
| 1. **Attending** the academic Half-Day has been … | **ease of attending** |
| 1. How **useful** is the content of the academic session to you … | **usefulness** |
| 1. How often, in your opinion, the material taught to you in class, **influences** your clinical decision making | **influence** |
| 1. How much does the content taught in the class, **add to/refresh** your knowledge | **refresh** |

**Appendix 3:** Qualitative Data from the Fellows who participated in the 2011–2013 Surveys on the Academic Half-Day Format

**2011 Survery:**

**Advantages**

- "[The] dedicated time for education [was] very valuable."
- "[The AHD format made it] easier to schedule the week."
- “When on services outside of MD Anderson Cancer Center, it is far easier to attend lectures in this format rather than in the old format of an hour lecture at lunch on Tuesdays and Thursdays.”
- “[The AHD advantages include] dedicated time for fellows to learn core principles and information; [the AHD format also] promotes opportunities for fellows to bond and exchange information.”
- “It is a protected block of time, and you don't have to fight everyday with attendings to leave for the lecture at noon; they know that this is our educational time.”
- “You get your didactic training in one day, which helps make the rest of the week flow better. In some ways, I preferred it this way.”

**Disadvantages**

- “Lecture fatigue”
- “[The AHD format] should continue with some improvement. Retention is difficult.”
- “Most of the lectures are didactic and not interactive, [which made it] very difficult to concentrate.”
- “Clinical responsibilities and carrying code pager during sessions”
- “4 hours of lectures is a little too long to tolerate and maintain a good attention span.”
- “Would be nice to have food!"
- “Learning environment. Gaps during the 4 hour block felt like idle time was being wasted.”

**Suggestions**

- “A core group of faculty with good general oncology knowledge should be present to help shape discussion and fine-tune fellows’ learning activity. Academic Tuesdays are currently unguided (i.e. no ‘Home base’ for fellows and core faculty). Academic Tuesdays could be that ‘home base.’”

**2012 Survey:**

**Advantages**

- "Pharmacology lectures are an excellent addition! Great way to learn oncology, and it has improved significantly since last year!"
- "Dedicated, relatively protected time to learn; camaraderie building"
- "This academic year is much better than last year. I really enjoy the protected time. If these were lunch hour lectures you would have inevitably been late or missed lectures owing to patient care. Lectures are now clinically relevant and geared toward the boards!”
- “Very well-organized”
- “Camaraderie with co-fellows, good lectures, food”
- “I liked having consolidated lectures; thank you for cramming more into the morning.”
- “I think it is great to have a half day format; keep it this way please.”
- “I liked having the opportunity to hear from multidisciplinary colleagues, especially pharmacology fellows.”

**Disadvantages**

- “Too little faculty involvement in journal club and Hong’s conference; not enough benign Heme lectures; too didactic; wish there were more interactive lectures”
- “Sometimes time is not managed well, and gaps are present between lectures. However, there has been a substantial positive improvement since last year.”
- “If a fellow misses one day, it is a lot of information missed; we need an electronic way of making it up (recorded lectures).”

**Suggestions**

- “Keep up the great work.”
- “Make ALL lectures available online. We could video-record sessions (especially didactic ones).”
- “More benign Heme. More question and answer sessions like board review, case scenarios, etc…”
- “Compilation of articles as references provided at each block. Written syllabus”

**2013 Survey:**

**Advantages**

- “Having disease blocks is fantastic to consolidate knowledge.”
- “It allows a block of time for educational activities. It is easy to attend because we are excused from clinical duties at this time. It helps build camaraderie. Posting Power Points online is helpful. The AHD format gives us access to faculty.”
- “Much better than having multiple 1-hour lectures on a daily basis, as this is our most protected time.”
- “Protected time, no awkward getting out of clinic early or late. Focused time”

**Disadvantages**

- “Many of the components of the Academic Tuesday are not very educationally high yield (especially journal club articles).”
- “Lack of innovative content and/or well-delivered and relevant content”
- “Not all lectures appear to be board relevant.”
- “We have to stay focused for a couple of hours, which is also challenging since completing medical school”
- “Not enough high-quality lectures. We did not get enough core-learning blocks over the past 12 months. The recording of the lectures hasn’t really added to my experience. I don’t think many fellows use this feature.”
- “Three hours of lecture in a row can be too much. My attention span wanes toward the end of the morning.”

**Suggestions**

- “None. Has gotten better over the years.”
- “ In an ideal world 🡪 no pages”
- “More interactive sessions, more small-group work. More varied types of sessions (not just powerpoint)”
- “12-month program. Increment faculty participation”

**2014 Survey:**

**Advantages**

- Having protected time for learning is an amazing opportunity to enrich our understanding. The PBL and interactive learning was an excellent supplement.”
- “Great lectures in a confined time frame. Great faculty presence this year.”
- “Great organization compared to previous year. Improvement getting 2 lectures.”
- “This year was much improved over last. Comprehensive coverage of important topics. Love the move to having fellow lectures over lunch.”
- “Time to concentrate on the knowledge base.”

**Disadvantages**

- “Sometimes difficult to hold concentration for 4 hours.”
- “Sometimes hard to digest such large blocks of information.”
- “Does take away time from research and sometimes need to multi-task to optimize efficiency.”
- “Slides are not always up-to-date after presentations.

**Suggestions**

- “More knowledge lectures. The “knots and bolts” of oncology rather than the latest and greatest research. We get exposure to the later in clinics, journal clubs, etc.
- “I like the current system as outlined. Continue sign in sheets instead of clickers as it is more reliable.”
- “Please provide caffeinated soda at lunch. It would make focusing on the noon content easier”.
- “I think the 12 months curriculum was a great change. Continue.”
- “Continued encouragement of academic leaders participating in lectures.”
- “Preferred 8-12 as opposed to 8-1 pm”
- May be bi weekly?? Don’t think we need this every week.”
- Having faculty present less on their research would help narrow the focus to the better. A summary slide of what you need to know for the boards would be helpful. More pharmacy talks for each disease type would be helpful.”

**Appendix 4:** Survey Reliability and Validity Data

**Survey Item generation:** Both authors: AE and PH designed the first year survey. After delivering the first survey, we conducted two focus group meetings consisting of fellows who had already graduated and current fellows, to validate survey items. The initial survey consisted of only four core items. The surveys from the first and second survey year were used for feasibility testing and further item generation. Four additional items were generated after reviewing the feedback from the first two years and input from faculty. The third survey in 2013 was used for validation. The core items from this validation survey consisted of – ease of organization, motivation to attend, ability to retain, ability to concentrate, distraction caused, usefulness, influence in clinical decision making, and refresh/addition to knowledge. These items were scores on a Likert scale with least favorable outcome at one end and the ideal outcome at the other end. A higher score indicated a favorable outcome. The third year survey was administered on paper. Additional questions that were asked on additional pages of the surveys to assist with the validation were the class of graduation, their future career goal, followed by specific items pertaining to the programs and methods implemented during the year, followed by a list of potential barriers experienced by the fellows and then finally a four question based cognitive debriefing survey. There were also qualitative questions (Appendix 1).

**Reliability:** The scale proved to be internally very consistent with a Cronbach's alpha of 0.83. Deleting one item at a time and recalculating the Cronbach’s alpha gave similar values as the full scale, indicating that each item contributed to the scale and should be retained in the scale.

**Validity:** Face and content validity of the first year survey were achieved through discussion among authors and review by attending physician colleagues. For construct validity we used the data from the 2013 survey with eight candidate items (Table A). Thirty four fellows responded to these items and there was no missing data. The scree plot yielded a two factor solution for the validation survey (Figure A). The test to see if such a two factor solution was sufficient was not rejected at a p-value of 0.549. Maximum likelihood factor analysis with varimax rotation was performed. The analysis yielded the two constructs having a cumulative variance of 0.53. Their factor loadings and factor analysis plot are presented in table B and figure B respectively. The items ‘concentration’, ‘organization’ and ‘distraction’ loaded closely on the first axis, while the other five items loaded closely on the second axis. Two constructs were identified. The first construct was identified as a “learning experience” domain and the second construct was identified as a “cognitive impact” domain. The scale proved to be internally very consistent with a Cronbach's alpha of 0.83. Deleting one item at a time and recalculating the Cronbach’s alpha gave similar values as the full scale (Table C), indicating that each item contributed to the scale and should be retained in the scale. The cognitive debriefing questions were also based on a Likert like scale with scores ranging from 1 to 5. Higher scores showed ease, comfort or comprehensiveness while lower scores showed discomfort or lacking. The results showed that the scale was easy to comprehend (4.5, sd-0.7), easy to complete (4.4, sd-0.7), comprehensive in inquiring about the academic learning program (3.75, sd-0.8) and the fellows felt comfortable in responding to the questions (4.4, sd-0.7).

**Using the Scale:** Table 2 in the manuscript shows the means and standard deviation of the scale items. The final validation scale shows that the various items measuring the fellow’s perception about our academic program range from 3.18 for the item “distractions caused” (reverse coded) to 4.26 for the item “motivation”. The overall mean for the entire scale was 3.78 which correspond close to the “agree” or “above average” anchor on our Likert scale. So, at an average our fellows found our program to be above average. It also identifies the areas that needed the most attention going forward. Distraction scored the lowest at 3.18, indicating that the fellows did feel quite distracted by various interruptions related to their duties. This is further discussed in the section below. Retention was also identified as another area needing future attention, with the score of 3.41, scoring lower that most other items. Looking at the means for the constructs we see that the “learning experience” domain scored an average of 3.61 and “cognitive impact” domain scored an average of 3.874. The four items, which were assessed during previous years, showed an improving trend from year 2011 to 2012 and maintained that trend in 2013. The reason for this improvement might be gauged from the improvements made to the academic half-day program. To evaluate the trend over the three years and six cohorts we did MANOVA with survey year and cohort as predictors and the four survey items as outcomes. We saw that the outcomes changed significantly over the three years and of them motivation and concentration were significant at 0.001 alpha level. This strongly suggest that the methods and format (like having a half-day format and protected time) applied over the three years has been improving and helping the fellows to concentrate and make them more motivated to attend the program.

**Table A: Means of candidate items from 2013 survey used for construct validation:**

| **Item** | **Mean (SD)** |
| --- | --- |
| Organization | 3.97 (0.80) |
| Motivation | 4.26 (0.86) |
| Retention | 3.41 (0.74) |
| Concentration | 3.68 (0.81) |
| Distraction | 3.18 (0.76) |
| Useful | 3.88 (0.73) |
| Influence | 3.76 (0.65) |
| Refresh | 4.06 (0.69) |

**Table B: Factor loadings**

|  | **Factor1** | **Factor2** |
| --- | --- | --- |
| Motivation | 0.61 | 0.47 |
| Useful | 0.9 |  |
| Influence | 0.84 |  |
| Refresh | 0.65 |  |
| Retention | 0.41 |  |
| Distraction |  | 0.7 |
| Organization |  | 0.65 |
| Concentration |  | 0.49 |

## Table C: Cronbach’s alpha

|  | **Cronbach’s alpha** |
| --- | --- |
| **Entire Scale** | **0.825** |
| **Item removed** |  |
| Organization | 0.835 |
| Motivation | 0.784 |
| Retention | 0.812 |
| Concentration | 0.812 |
| Distraction | 0.806 |
| Useful | 0.793 |
| Influence | 0.804 |
| Refresh | 0.788 |

**Figure A: Scree Plot**


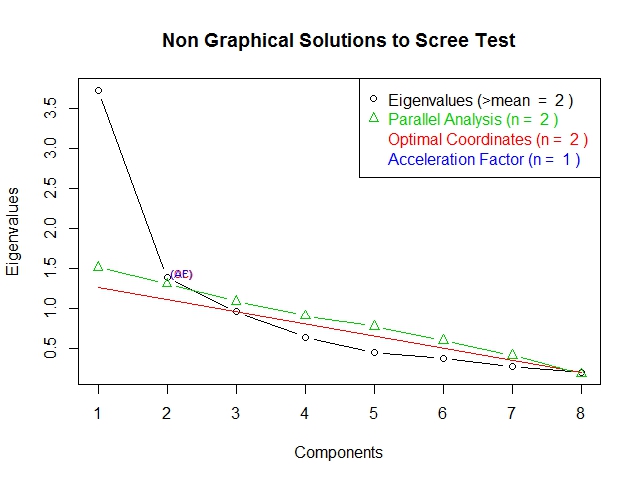


**Figure B: Factor Analysis**


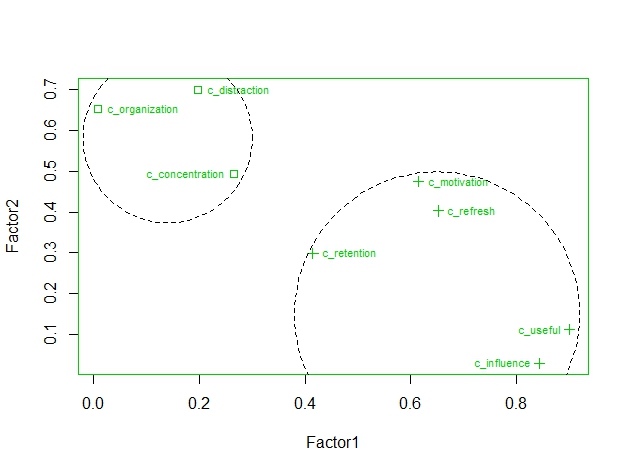

Supplement: Additional file 1: — Appendices 1 to 4. (DOCX 305 kb) [file 12909_2015_418_MOESM1_ESM.docx]
